# Effort-Reward Imbalance at School and Depressive Symptoms in Chinese Adolescents: The Role of Family Socioeconomic Status

**DOI:** 10.3390/ijerph110606085

**Published:** 2014-06-10

**Authors:** Hongxiang Guo, Wenjie Yang, Ying Cao, Jian Li, Johannes Siegrist

**Affiliations:** 1 Department of Pediatrics, the First Affiliated Hospital of Zhengzhou University, Zhengzhou 450052, China; E-Mail: doctordoris@sina.com; 2 School of Public Health, Zhengzhou University, Zhengzhou 450001, China; E-Mail: ywjie@zzu.edu.cn; 3 First High School of Zhengzhou, Zhengzhou 450000, China; E-Mail: zz.caoying@gmail.com; 4 Department of Education, Tufts University, Medford, MA 02155, USA; 5 Institute of Occupational and Social Medicine, Centre for Health and Society, Faculty of Medicine, University of Düsseldorf, Universitätsstrasse 1, Düsseldorf 40225, Germany; 6 Senior Professorship on Work Stress Research, Life-Science Centre, University of Düsseldorf, Düsseldorf 40225, Germany; E-Mail: johannes.siegrist@med.uni-duesseldorf.de

**Keywords:** school-related stress, effort-reward imbalance, socioeconomic status, depression, adolescents, China

## Abstract

Depression is a major mental health problem during adolescence. This study, using a sample of Chinese adolescents, examined the separate and combined effects of perceived school-related stress and of family socioeconomic status (SES) on the prevalence of depressive symptoms. A total of 1774 Chinese students from Grades 7–12 were recruited into our questionnaire survey. School-related stress was measured by the Effort-Reward Imbalance Questionnaire-School Version, family SES was assessed by a standardized question, and depressive symptoms were evaluated by the Center for Epidemiological Studies Depression Scale for Children. Multivariate logistic regression was applied, adjusting for age, gender, grade, smoking, alcohol drinking and physical activity. It was found that high school-related stress and low family SES were associated with elevated odds of depressive symptoms, respectively. The effect of school-related stress was particularly strong in low SES group. In adolescents with both high stress at school and low SES, the odds ratio was 9.18 (95% confidence interval = 6.53–12.89) compared to the reference group (low stress at school and high SES). A significant synergistic interaction effect was observed (synergy index = 2.28, 95% confidence interval = 1.56–3.32). The findings indicated that perceived school-related stress, in terms of effort-reward imbalance, was related to depressive symptoms in this sample of Chinese adolescents. The strong interaction with family SES suggests that health promoting efforts in school settings should be targeted specifically at these socially deprived groups.

## 1. Introduction

Schools are an important psychosocial environment of adolescents. This is not only due to the fact that performance at school largely determines academic achievement, but also because schools provide the main broader social arena of recurrent interpersonal exchange of adolescents in formal (with teachers) and informal (with schoolmates) relationships. Experiences of success or failure and of favorable or unfavorable social comparison processes exert strong effects on adolescents’ self-confidence and their mental well-being, especially so during this sensitive period of psychosocial and biological change [1]. Several studies document associations of stressful experience at school with reduced psychosomatic health and well-being [2,3,4,5,6,7,8,9,10,11,12,13,14]. Yet, these studies rarely defined school environments in a stress-theoretical framework, thus preventing the comparison and generalization of respective findings [5,6,15]. Moreover, few investigations focused on depressive symptoms [8,9,12,14]. Despite a somewhat milder condition compared to major depressive episodes, depressive symptoms are remarkably prevalent during adolescence, and they were shown to exert severe negative effects on psychopathology and educational impairments [16,17].

In this study we set out to add three innovative elements to the current state of research in this context. First, we analyze the association of stressful experience at school with depressive symptoms in the frame of a theoretical model, Effort-Reward Imbalance. This model posits that failed reciprocity between high effort spent and low reward received in turn adversely affects the health of acting people [18]. Importantly, reward includes status-related gains as well as esteem or recognition. While this model was primarily applied to explain work-related health risks among employed adults [19,20,21,22,23,24], it was more recently tested with respect to other forms of performance-based social exchange, such as volunteering [25], obligations between parents and children [26], and achievement-related exchange in school settings [27]. To this latter end, a specific questionnaire was developed and psychometrically validated to assess students’ experiences of perceived psychosocial stress at school [28].

Second, we additionally explore the role of family socioeconomic status (SES) in analyzing associations of school-related stressful experience with depressive symptoms. It is well known that adolescents with low SES exhibit elevated risks of suffering from depression compared to those of adolescents with a more favorable social background [29,30,31,32,33,34,35,36,37]. While an explanation of this link cannot be advanced in the frame of a cross-sectional study it seems nevertheless justified to test the assumption that family SES moderates this association, such that stronger associations of school-related stress with depressive symptoms are observed among adolescents with a disadvantaged social background. This assumption points to their increased susceptibility to stressful experience due to limited coping resources and increased exposure to interpersonal conflicts and harassment [38,39,40,41].

Third, while a majority of investigations on the associations of school environments with adolescent health were conducted in high income countries, respective research in rapidly developing countries is badly needed, given the public health burden of mental disorders in low and middle income countries [42,43,44]. China is one such important rapidly developing country. Moreover, in traditional Chinese culture, school is considered a particularly stressful setting, where students are under high academic burden and pressure due to high expectations of their parents and strong competition with their peers [28,45]. Recent investigations addressing contemporary Chinese society indicate a social gradient of adolescent health, leaving those with lower SES in poorer health [35].

Based on these arguments we test associations of school-related stress in terms of effort-reward imbalance with depressive symptoms in a large sample of male and female Chinese students of mid to late adolescence, with a special emphasis on the moderating role of family SES.

## 2. Methods

### 2.1. Sample

A cross-sectional study was conducted in Zhengzhou, the capital city of Henan Province in central China. Two schools (one middle school with grades 7–9 and one high school with grades 10–12) located in central areas of the city were invited and both agreed to participate in our questionnaire survey. In these two schools, each grade consisted of 10 classes. We randomly selected five classes from every grade, resulting in a total of 30 classes. According to the International Standard Classification of Education (ISCED) by the United Nations Educational, Scientific and Cultural Organization (UNESCO) [46], grades 7 through 9 belong to level 2 (lower secondary education) which is still compulsory in China; grades 10 through 12 belong to level 3 (upper secondary education) which is no longer compulsory in China. A total of 2035 students from grades 7 to 12 were invited to participate. The questionnaire was returned by 1803 students (response rate = 88.60%) who were recruited from a total of 30 classes. This current report is based on a sample of 1774 students whose questionnaires had no missing values. This study was approved by the Ethical Committee of the Zhengzhou University, and was performed in accordance with the Declaration of Helsinki.

### 2.2. Measures

#### 2.2.1. Perceived School-Related Stress

This main independent variable was measured by a psychometrically validated questionnaire, “Effort-Reward Imbalance at School” (ERI-S) [28], which consists of two scales, “effort” (five items addressing schoolwork and related expectations; Cronbach’s alpha coefficient = 0.73 in this study) and “reward” (three items measuring academic performance, seven items assessing esteem, and one item indicating study prospects; Cronbach’s alpha coefficient = 0.73 in this study). The items were answered on a five-point Likert scale, ranging from “strongly disagree” to “strongly agree”. According to a predefined algorithm, a ratio between the two scales “effort” and “reward” (weighted by item numbers) was calculated to quantify the degree of mismatch between high “cost” and low “gain”, as proposed by the original test author [18], and a dichotomized effort-reward ratio with a cut-point > 1.0 was defined as a high stress condition [47].

#### 2.2.2. Family SES

Family SES was measured by a single question: “How well off do you think your family is?” The response categories were “very well off”, “quite well off”, “average”, “not very well off”, “not all well off”. Adolescents rating their family were “very well off” or “quite well off” were classified as group with high family SES, otherwise with low family SES. This one question on family SES has been widely used in European and American young students based on the World Health Organization (WHO) collaborative cross-national survey of Health Behaviour in School-aged Children (HBSC) [48,49], and it was recently applied in the Chinese context, showing considerably high correlations to objective measures of adolescent SES, such as Family Affluence Scale and variables assessing parents’ educational levels [50].

#### 2.2.3. Depressive Symptoms

The Center for Epidemiological Studies Depression Scale for Children (CES-DC) has been used to screen for depressive symptoms and disorder in children and adolescents for more than two decades [51], we applied a validated Chinese version of this scale [52]. It consists of 20 self-report items answered by a four-point response option (0 = not at all, 1 = a little, 2 = some, 3 = a lot). Thus, the total score ranges from 0 to 60, with higher scores reflecting higher depressive symptoms. Cronbach’s alpha coefficient was 0.89 in our survey. According to previous research conducted among Chinese adolescents, CES-DC score greater than 28 was considered to indicate depressive symptoms in this study [12,53].

In addition, information on age, gender, grade, smoking, alcohol drinking, and physical activity was also collected. These latter variables were included as potential confounders in multivariate regression models as they might be associated with school environment [44], as well as with socioeconomic status [41] and depression during adolescence [16].

### 2.3. Statistical Analysis

Following descriptive statistics we applied *t*-test (for continuous variables) or Chi-square test (for categorical variables) to compare the differences between the two groups with high and low family SES. Subsequently, multivariate logistic regression models were calculated to test the hypotheses. Results are displayed as odd ratios (ORs) with 95% confidence intervals (CIs). In stepwise regression models ORs were adjusted for relevant confounders. We confirmed the fit of the logistic regression models with the Hosmer-Lemeshow goodness-of fit test. In all cases, appropriate model fits of the data were observed (*p* > 0.05).

First, associations between school-related stress, family SES, and depressive symptoms were examined using the total sample, whilst school-related stress and family SES were adjusted for each other. Next, we explored associations of school-related stress with depressive symptoms stratified by family SES. Additionally, we examined these associations for effort, reward, and three sub-components of reward, *i.e.*, academic performance, esteem, and study prospects. Given the fact that all these variables were measured as continuous data, the ORs were expressed as increase by one standard deviation (SD). Finally, to test the interaction of school-related stress and family SES, a composite variable with different combinations was constructed: (1) low school-related stress and high family SES; (2) low school-related stress and low family SES; (3) high school-related stress and high family SES; (4) high school-related stress and low family SES. The synergy index and 95% CI were calculated to examine the joint effects of school-related stress and family SES. Results may indicate synergistic interaction (synergy index > 1), additive interaction (synergy index = 1), or antagonistic interaction (synergy index < 1) [54]. Analyses were performed with the statistical program SAS 9.2 (SAS Institute Inc., Cary, NC, USA).

## 3. Results

Table 1 shows the main characteristics of the study population. In total, there were 993 boys and 781 girls with an average age of 16 years. Among them, 34.27% came from low SES families. More than 800 students experienced high school-related stress measured by effort-reward imbalance (45%). The overall prevalence of depressive symptoms was 24%. We did not find significant gender differences in the distribution of family SES, school-related stress and prevalence of depressive symptoms (data not shown). When comparing the students from different SES families, the students with low SES were significantly older and smoked more frequently. The prevalence rates of school-related stress and depressive symptoms were significantly higher among adolescents from low SES families than adolescents from high SES families (54% *vs.* 41%, 33% *vs.* 19%, respectively). This holds particularly true for high effort and three sub-components measuring low reward.

In Table 2, the associations of school-related stress and family SES with depressive symptoms are displayed. In the unadjusted model, high school-related stress (including the components of Effort-Reward Imbalance model) and low family SES were significantly associated with depressive symptoms, respectively. Adjusting for socio-demographic and health-related behaviors (models II and III) did not substantially reduce the size of the ORs. 

With the stratified analyses by family SES, the associations between high school-related stress and depressive symptoms were consistent throughout different SES groups, but the effects in low SES group were stronger.

**Table 1:** Characteristics of the study subjects.

Variables		High Family SES (N= 1166)	Low Family SES (N = 608)	*p*
Age (mean ± SD)		15.88 ± 1.53	16.13 ± 1.54	0.0010
Gender (N; %)	Boys	659; 56.52%	334; 54.93%	0.5236
	Girls	507; 43.48%	274; 45.07%	
Grades (N; %)	Grades 7–9	609; 52.23%	287; 47.20%	0.3540
	Grades 10–12	557; 47.77%	321; 52.80%	
Smoking (N; %)	No	1142; 97.94%	582; 95.72%	0.0074
	Yes	24; 2.06%	26; 4.28%	
Alcohol drinking (N; %)	No	945; 81.05%	484; 79.61%	0.4667
	Yes	221; 18.95%	124; 20.39%	
Physical inactivity (N; %)	Active	339; 29.07%	153; 25.16%	0.0809
	Inactive	827; 70.93%	455; 74.84%	
School-related stress (N; %)	Low (Effort-Reward ratio < = 1)	687; 58.92%	281; 46.22%	<0.0001
	High (Effort-Reward ratio > 1)	479; 41.08%	327; 53.78%	
Effort (mean ± SD)		17.44 ± 3.46	18.18 ± 3.30	<0.0001
Reward (mean ± SD)		40.74 ± 5.23	38.57 ± 5.62	<0.0001
Academic performance (mean ± SD)		10.25 ± 2.10	9.59 ± 2.03	<0.0001
Esteem (mean ± SD)		26.59 ± 4.04	25.29 ± 4.51	<0.0001
Study prospects (mean ± SD)		3.90 ± 0.92	3.69 ± 1.00	<0.0001
Depressive symptoms (N; %)	No	942; 80.79%	405; 66.61%	<0.0001
	Yes	224; 19.21%	203; 33.39%	

Note: Differences were determined by Student’s *t*-test or Chi-square test.

**Table 2:** Associations of school-related stress and family SES with depressive symptoms (ORs and 95% CIs).

Total sample		Model I	Model II		Model III
School-related stress	Low	1	1		1
	High	5.38 (4.19, 6.91) ***	5.34 (4.15, 6.88) ***		5.16 (4.00, 6.65) ***
Effort	Increase per SD	2.03 (1.76, 2.34) ***	2.03 (1.76, 2.35) ***		2.02 (1.74, 2.33) ***
Reward	Increase per SD	0.43 (0.37, 0.49) ***	0.43 (0.37, 0.49) ***		0.43 (0.37, 0.50) ***
Academic performance	Increase per SD	0.56 (0.49, 0.64) ***	0.54 (0.47, 0.62) ***		0.54 (0.48, 0.62) ***
Esteem	Increase per SD	0.52 (0.46, 0.59) ***	0.52 (0.45, 0.59) ***		0.52 (0.46, 0.60) ***
Study prospects	Increase per SD	0.78 (0.70, 0.88) ***	0.78 (0.70, 0.88) ***		0.78 (0.70, 0.88) ***
Family SES	High	1	1		1
	Low	1.86 (1.46, 2.35) ***	1.84 (1.45, 2.33) ***		1.84 (1.45, 2.34) ***
		**High family SES sample (fully adjusted model)**		**Low family SES sample (fully adjusted model)**	
School-related stress	Low	1		1	
	High	4.12 (2.99, 5.69) ***		7.62 (4.98, 11.66) ***	
Effort	Increase per SD	1.92 (1.59, 2.32) ***		2.20 (1.74, 2.78) ***	
Reward	Increase per SD	0.46 (0.39, 0.55) ***		0.39 (0.31, 0.50) ***	
Academic performance	Increase per SD	0.58 (0.47, 0.71) ***		0.51 (0.43, 0.61) ***	
Esteem	Increase per SD	0.57 (0.48, 0.67) ***		0.47 (0.38, 0.58) ***	
Study prospects	Increase per SD	0.84 (0.72, 0.98) *		0.69 (0.57, 0.83) ***	

Note: Logistic regression, * *p* < 0.05, *** *p* < 0.001; Model I: non-adjustment; Model II: adjustment for age, gender, and grade; Model III: Model II + additional adjustment for smoking, alcohol drinking, and physical activity.

**Figure 1:**
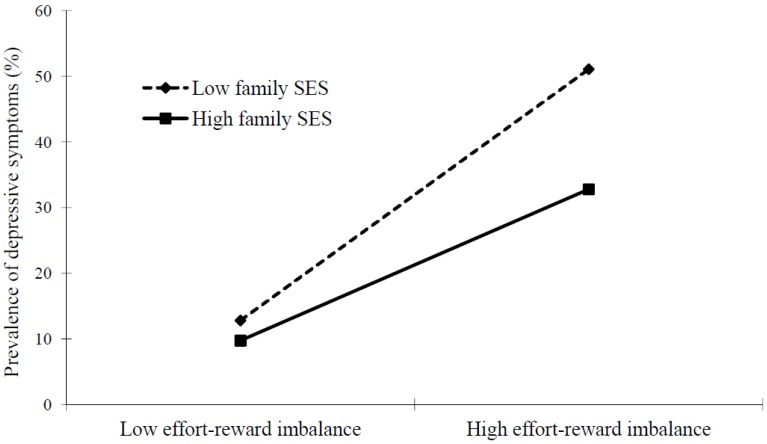
Two-way moderation of school-related stress, family SES and depressive symptoms.

The interaction analyses indicated that family SES significantly moderated the association between school-related stress measured by effort-reward imbalance and depressive symptoms (Figure 1). The odds of depressive symptoms were markedly elevated if adolescents exhibited high school-related stress and high family SES, or high school-related stress and low family SES simultaneously (OR = 4.26 with 95% CI = 3.10–5.87, OR = 9.18 with 95% CI = 6.53–12.89, respectively), and the synergistic effect was highly significant, in the fully adjusted model (Table 3).

**Table 3:** Joint effects of school-related stress and family SES on depressive symptoms (ORs and 95% CIs).

School-related stress	Family SES	Model I	Model II	Model III
Low	High	1	1	1
Low	Low	1.36 (0.88, 2.09)	1.33 (0.86, 2.05)	1.32 (0.86, 2.04)
High	High	4.51 (3.29, 6.19) ***	4.45 (3.24, 6.12) ***	4.26 (3.10, 5.87) ***
High	Low	9.66 (6.92, 13.46) ***	9.48 (6.77, 13.29) ***	9.18 (6.53, 12.89) ***
Synergy index		2.24 (1.55, 3.22) ***	2.24 (1.55, 3.25) ***	2.28 (1.56, 3.32) ***

Note: Logistic regression, *** *p* < 0.001; Model I: non-adjustment; Model II: adjustment for age, gender, and grade; Model III: Model II + additional adjustment for smoking, alcohol drinking, and physical activity.

## 4. Discussion

This study adds three new elements to the current state of research on the impact of school-related stress on adolescent mental health. First, we documented robust associations of perceived experience of stress at school, as measured by the Effort-Reward Imbalance model, with depressive symptoms, independent of age and gender. Second, a strong moderation effect of family SES was demonstrated, such that associations of school-related stress with depressive symptoms were substantially stronger among adolescents with low SES compared to those of adolescents with a more favorable socioeconomic background. Third, the results were derived from a large sample of adolescents in China, a rapidly developing country where school is considered a particularly stressful setting.

Why is stressful experience at school more harmful in adolescents with low SES? According to a leading hypothesis in this field of research, families with low SES are less capable to provide those material and psychosocial resources that strengthen their children’s coping abilities, compared to families who live in more privileged social positions [1]. Therefore, these adolescents are more likely to be exposed to recurrent stressful encounters in the school environment, a significant setting where young people spend a majority of their time. In addition, the fact that adolescents with low SES background have significantly lower academic performance adds to the burden of their social disadvantage. Thus, they receive less esteem from teachers and schoolmates. Unfavorable social comparison processes and inappropriate coping skills may impair their self-esteem and render them more vulnerable to the experience of striving without success. This experience, in turn, may increase their susceptibility to depressive symptoms [39,55]. Moreover, some studies suggested that adolescents with low SES had limited access to healthcare [56,57]. With respect to the influence of parents’ health and the psychosocial pressure they exert, recent research has shown that the intergenerational transmission of mental health problems is particularly strong in families of low SES [33]. Concerning the latter aspect, Chinese parents in general have high expectations concerning their only-one child’s academic achievement, thereby increasing the stress burden on their child [13,45].

Several limitations of our study have to be addressed. First, given the cross-sectional design and the self-reported measures of all variables, common method variance might bias the associations in this study [58]. We cannot exclude that depressive mood affects the degree to which the school is experienced as stressful. However, evidence from prospective cohort studies of working populations indicates that effort-reward imbalance clearly precedes the development of depressive episodes [19,21,22,23,24]. Second, the validity of our measurement of SES during adolescence may be limited, given its summary estimate and given substantial fluctuations of the socioeconomic standing of families in the current context of rapid economic development in this country [30]. A more detailed measure of SES proposed by the WHO-HBSC study group, the Family Affluence Scale for researching health inequalities in adolescents [59] was not available in this study. Third, some risk factors which have been identified as stressors during adolescence in previous studies, such as negative life events and bullying [60], were not included in this current investigation. Fourth, with its focus on stressful experience at school, our study addressed the meso-level of potential determinants of adolescent depressive symptoms. More recently, some studies highlighted the importance of micro-level and personal risk factors of depression, in particular genetic susceptibility, thereby pointing to the direction of exploring gene-environment interactions [61,62]. Finally, despite the large sample size included in this study, the results are restricted to two selected schools of a large city in one country. Clearly we cannot claim that our findings can be generalized to school settings in other locations and regions within and beyond China.

These limitations are balanced by several strengths. In terms of methodology, core variables, such as perceived school-related stress and depressive symptoms, were measured with theory-based, psychometrically validated instruments. Moreover, we conducted a rigorous statistical test of the moderation hypothesis which, to our knowledge, has not yet been explored in adolescent populations. Our findings support the notion of a social gradient of reduced mental health in adolescents within a rapidly developing country of considerable global significance. In addition, by demonstrating the increased vulnerability of adolescents with low SES who are exposed to school-related stress, the results of this study suggest that preventive efforts of strengthening mental health at school should be directed primarily towards this latter group [63]. In future, more well-designed longitudinal studies including more schools from diverse socioeconomic strata are warranted, investigating deeply on stressful experience at school and social inequalities in adolescent mental health.

## 5. Conclusions

In this study, perceived school-related stress as measured by the Effort-Reward Imbalance model was significantly associated with depressive symptoms in a large sample of Chinese adolescents, and this association was particularly strong among adolescents from families with low SES. Our findings suggest that theory-based health promotion programs in school settings should be developed and should pay special attention to adolescents exposed to school-related stress, in particular, to socially deprived groups.
